# Treatment of facial lipodystrophy induced by a biologic agent (IPD-1): a literature review

**DOI:** 10.31744/einstein_journal/2024RC1111

**Published:** 2024-11-06

**Authors:** Henri Friedhofer, Cristina Pires Camargo, Leandro Hirokazu Oshiro, Daniel de Almeida Rocha Valente, Rolf Gemperli

**Affiliations:** 1 Universidade de São Paulo Faculdade de Medicina Hospital das Clínicas São Paulo SP Brazil Division of Plastic Surgery, Hospital das Clínicas, Faculdade de Medicina, Universidade de São Paulo, São Paulo, SP, Brazil.

**Keywords:** Carcinoma, renal cell, Nivolumab, Thyroid gland, T-Lymphocytes, Diabetes mellitus, Lipodystrophy, Adipose tissue, Apoptosis

## Abstract

Agents that inhibit programmed cell death (IPD-1) in T lymphocytes are indicated for patients with advanced cancer. However, some individuals may develop endocrinological conditions, such as diabetes, thyroid dysfunction, and lipodystrophy, after treatment. This systematic review and case report of IPD-1 lipodystrophies describes a patient who received nivolumab treatment for advanced clear cell renal carcinoma and subsequently developed diabetes as well as facial and body lipodystrophy. The patient complained of social distress due to her facial appearance. We treated the facial lipodystrophy with autologous fat grafting, which proved to be effective for more than three years. This study showed the efficacy of IPD-1 lipodystrophy treatment with long-term follow-up.

## INTRODUCTION

Certain cancers develop immune resistance owing to immune checkpoint dysregulation. Although many pathways can be involved in immune resistance, T cell immunoregulatory therapy is one of several methods used to block cancer progression. T cells recognize tumor peptides in all cell compartments. T cell therapies involving CD8+ T cells, cytotoxic T lymphocytes, and CD4+ helper cells can be utilized to recognize and directly neutralize cancer cells.^(1,2)^ Therefore, T-cell therapy with anti-programmed death receptor 1 (IPD-1) is a plausible alternative for treating resistant cancer.^(2,3)^

T cell therapies have been used to treat advanced melanoma as well as renal and colorectal cancers with successful therapeutic outcomes. Nonetheless, these biological agents have occasionally been associated with several adverse events owing to autoimmune responses, such as hypophysis as well as thyroid and adrenal dysfunction. Rarely, self-immune reactions can destroy adipose tissue, leading to severe lipodystrophy.^(3-7)^

This study reviewed lipodystrophy caused by IPD-1 treatment and reported on facial lipodystrophy treated with fat grafting.

## CASE REPORT

A 57-year-old female patient was diagnosed with locally advanced clear-cell renal carcinoma of the left kidney in 2010. Therefore, she received two cycles of sunitinib (Sutent^®^, Pfizer, Brazil). Four months later, the patient underwent left nephrotomy and adrenalectomy. Four years after surgery, during a routine checkup, cancer progression was detected in the contralateral kidney, pancreas, liver, and adrenal gland. In December 2014, the patient began treatment with pazopanib (Votrient^®^, GlaxoSmithKline, Brazil); however, in the fifth chemotherapy cycle, she developed liver toxicity. Due to hepatic metastasis, surgery was recommended, including partial right nephrectomy and caudal pancreatectomy. Cytopathological examination revealed residual cancer cells in the right kidney, and after one year, cancer progression was noted in the right kidney and adrenal gland. Nivolumab (Opdivo^®^, Bristol Myers Squibb, Brazil) at a dosage of 3mg/kg was then prescribed. However, the treatment was suspended after 16 cycles owing to grade 3 hepatotoxicity.

In 2017, after the final cycle of nivolumab, the patient developed severe body and facial lipodystrophy.^(8)^ She was referred to the oculoplastic group, plastic surgery division, HC-FMUSP, for treatment. She presented severe grade IV (Facial Lipodystrophy Index) lipodystrophy, which had a negative impact on her social and professional life due to her appearance. Skin biopsy revealed chronic panniculitis and fibrosis. In 2018, after discussing this case with the oncologists, the surgical team decided to perform facial fat grafting.

This study was approved by *Hospital das Clínicas, Universidade de São Paulo (HC-FMUSP),* (CAAE: 66249022.3.0000.0068; #5.836.591). The patient provided written informed consent. The SCARE checklist was used.^(8)^

### Surgical procedure

**Figure 1:**
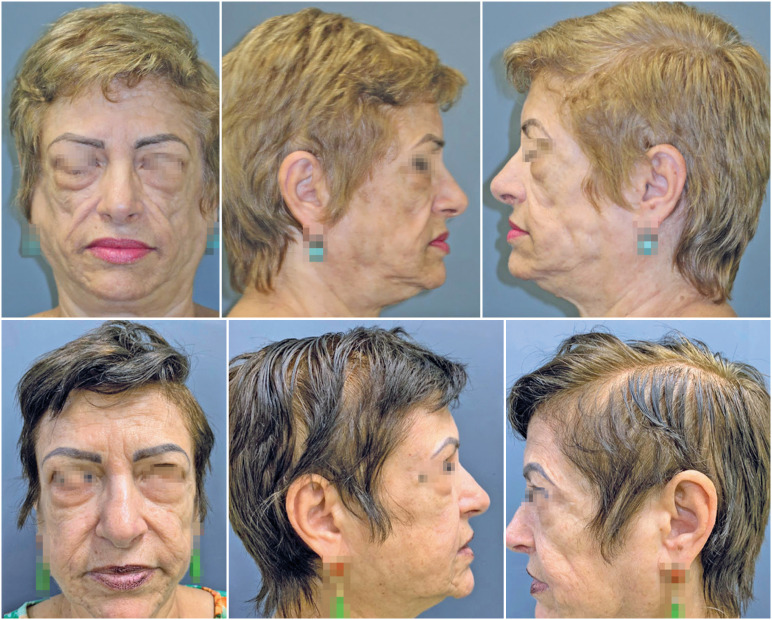
Comparison photographs showing the patient prior to the fat graft procedure (top row) and at the six-year postoperative follow-up (bottom row)

The patient was placed in the supine position and an anesthetic solution was administered in the infraumbilical region using a combination of lidocaine 2% (Xylesin, Cristalia, Brazil), epinephrine 1:1000 (Efrinalin, Blau Farmaceutica, Brazil), and 0.9% saline (Eurofarma, Brazil). After 10 minutes, 100mL of fat was suctioned using a 3mm liposuction cannula and 10mL syringes (Seringa, BD, Brazil). The syringe contents were decanted for 20 min, and the lower liquid phase was discarded. The remaining fat was washed three times with 0.9% saline solution and emulsified in a closed transfer system.^(9)^ Four punctures were made using a 40 × 12 needle (BD, Brazil), and 50mL of fat was injected (orbital margin, SOOF, malar region). The patient was prescribed cefazolin (1g TID) for seven days as postoperative care. After three years of follow-up, the patient continued to show satisfactory results (Figure 1).

### Literature review

We included all types of studies. The inclusion criteria were studies reporting adults (aged >18 years) who received at least one IPD-1 treatment and subsequently developed lipodystrophy.

This review analyzed the IPD-1 regimen, time, lipodystrophy region, and treatment. The characteristics of the studies are presented in table 1.

**Table 1:** Characteristics of the main literature studies

Author, year	Patient (sex, age - years)	Type of cancer	Cancer treatment	Lipodystrophy area
Drexler et al,^(10)^ 2021	Female, 41	Melanoma, T-III	Nivolumab 240mg, every two weeks, 12 months	Face, trunk, upper limbs
Haddad et al,^(11)^ 2020	Female, 47	Melanoma, T-IV	Pembrolizumab 2mg/kg, every 3 weeks, 10 weeks	Face, pigmentation disorder, hirsutism
Tjarks et al,^(12)^ 2018	Male, 61	Renal cancer	Nivolumab, 4 cycles	Trunk and limbs
Jehl et al,^(13)^ 2019	Female, 62	Melanoma	Nivolumab, 18 months, interrupted by hepatoxicity	Face, upper limbs, trunk
Bedrose et al,^(14)^ 2020	Male, 67	Melanoma, 4	Nivolumab, 6 weeks/Pembrolizuma, 4 months	Face and trunk
Gnanendran et al,^(15)^ 2020	Female, 34	Melanoma T3A	Nivolumab 3mg/kg, every 2 weeks, 9 months	Face and limbs
Bhargava et al,^(16)^2020	Female, 59	Melanoma	Nivolumab	Thigh(with necrosis)
Pach et al,^(17)^2021	Female, 78	Melanoma	Nivolumab, 10 months	Trunk and limbs

None of the studies treated the lipodystrophy region.

## DISCUSSION

This case report describes a straightforward surgical procedure for the treatment of IPD-1-induced facial lipodystrophy.

Lipodystrophy syndrome, with several etiologies and severity levels, can be either congenital or acquired. Acquired lipodystrophy can affect restricted areas of the body. The most common form of drug-induced lipodystrophy is associated with HIV anti-retroviral treatment.^(18-20)^ Endocrinological disorders causing lipodystrophy have also been described. Lipodystrophy aggravates insulin-related metabolism by compromising homeostasis.^(12)^

IPD-1-induced lipodystrophy is a recently described, drug-related etiology. However, given that lipodystrophy is uncommon, determining the time of onset or establishing a metabolic imbalance based on the severity of the lipodystrophy is not usually possible.^(3,4)^ Another point to consider is the negative impact of lipodystrophy on body image. Similar to several previously described cases, our patient was referred due to social and professional distress while performing her daily activities.^(21)^

Currently, no treatment exists for IPD-1 lipodystrophy, and to the best of our knowledge, this is the first report describing the use of fat grafting for the treatment of facial IPD-1 lipodystrophy. IPD-1 therapy has been used in the past to treat oncologically advanced disease; however, the side effects can be severe, and the negative impact on the daily lives of patients must be considered. In this study, our clinical team assessed the benefits and risks of the fat graft procedure.

Liposuction and fat grafting are minor surgeries, and in our case, the lower abdominal donor site had sufficient fat that could be harvested for the procedure. Surprisingly, the postoperative fat volume retention rate was satisfactory, even after long-term follow-up. The fat preparation procedure (decantation, washing, and fractionation) can increase the fat retention rate; however, further investigations are required to increase the level of certainty.

In this case report, we aimed to demonstrate a minimally invasive procedure (fat grafting) for the treatment of IPD-1-induced facial lipodystrophy.

## CONCLUSION

Our results suggest that this noninvasive surgery can improve the quality of life of patients presenting with facial IPD-1 lipodystrophy.
